# Two New FRET Imaging Measures: Linearly Proportional to and Highly Contrasting the Fraction of Active Molecules

**DOI:** 10.1371/journal.pone.0164254

**Published:** 2016-10-25

**Authors:** Masataka Yamao, Kazuhiro Aoki, Naoto Yukinawa, Shin Ishii, Michiyuki Matsuda, Honda Naoki

**Affiliations:** 1 Graduate School of Information Science, Nara Institute of Science and Technology, Ikoma, Nara, Japan; 2 National Institute for Basic Biology, Okazaki, Aichi, Japan; 3 Okinawa Institute of Science and Technology Graduate University, Kunigami, Okinawa, Japan; 4 Imaging Platform for Spatio-temporal Information, Kyoto University, Sakyo, Kyoto, Japan; 5 Graduate School of Informatics, Kyoto University, Sakyo, Kyoto, Japan; 6 Graduate School of Medicine, Kyoto University, Sakyo, Kyoto, Japan; Consiglio Nazionale delle Ricerche, ITALY

## Abstract

We developed two new FRET imaging measures for intramolecular FRET biosensors, called linearly proportional (LP) and highly contrasting (HC) measures, which can be easily calculated by the fluorescence intensities of donor and acceptor as a ratio between their weighted sums. As an alternative to the conventional ratiometric measure, which non-linearly depends on the fraction of active molecule, we first developed the LP measure, which is linearly proportional to the fraction of active molecules. The LP measure inherently unmixes bleed-through signals and is robust against fluorescence noise. By extending the LP measure, we furthermore designed the HC measure, which provides highly contrasting images of the molecular activity, more than the ratiometric measure. In addition to their advantages, these measures are insensitive to the biosensor expression level, which is a fundamental property of the ratiometric measure. Using artificial data and FRET imaging data, we showed that the LP measure effectively represents the fraction of active molecules and that the HC measure improves visual interpretability by providing high contrast images of molecular activity. Therefore, the LP and HC measures allow us to gain more quantitative and qualitative insights from FRET imaging than the ratiometric measure.

## Introduction

**Fig 1:**
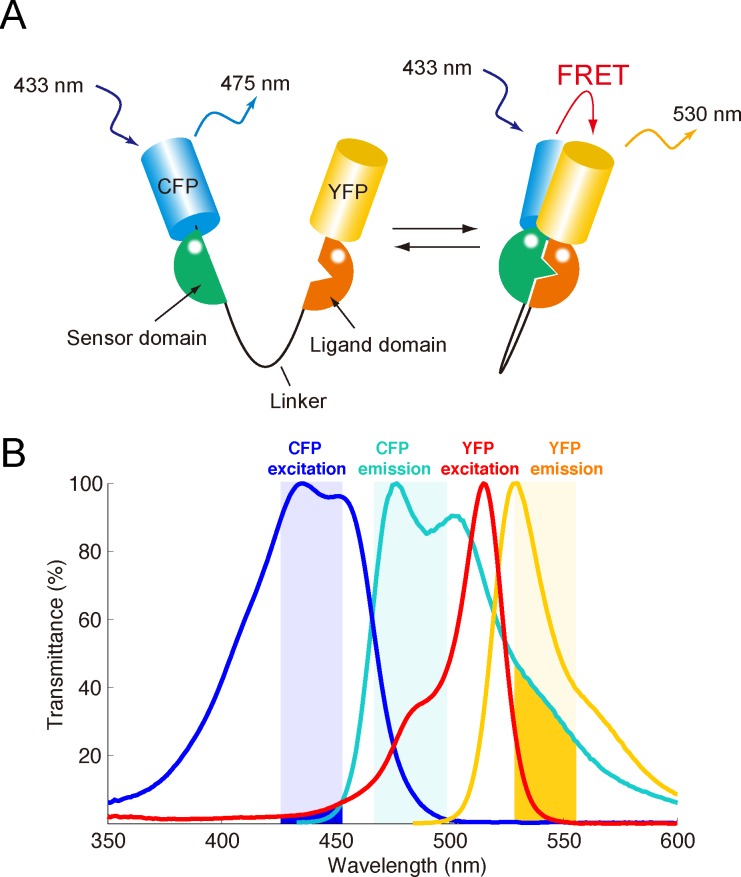
FRET biosensor. **(A)** Schematic of the structure of the intramolecular FRET biosensor, which consists of the studied protein (sensor domain) fused with a ligand domain, which is sandwiched by the donor and acceptor fluorophores, e.g., cyan fluorescent protein (CFP) and yellow fluorescent protein (YFP). The distance between the donor and the acceptor depends on the conformation, *i*.*e*., active or inactive state, of the focused protein. In the inactive state, the CFP is excited and emits fluorescence with a specific wavelength, whereas in the active state, FRET occurs due to the close proximity between the CFP and the YFP, and the YFP emits fluorescence with a different wavelength. **(B)** Normalized excitation and fluorescence spectra of the donor and acceptor fluorophores are shown. The CFP emission spectrum largely overlaps with the YFP excitation, enabling FRET. The blue, cyan and yellow shaded regions indicate the light wavelengths for CFP excitation, CFP emission filtering and YFP emission filtering, respectively. The blue and yellow regions represent cross-excitation and bleed-through.

To visualize the spatiotemporal dynamics of molecular activity in living organisms, biosensors have been developed based on the principle of Förster (or fluorescence) resonance energy transfer (FRET) [1–3]. Among them, genetically encoded, *i*.*e*., fluorescent protein-based, intramolecular FRET biosensors have been the most widely used due to the simplicity of the imaging. The intramolecular FRET biosensor emits fluorescence with wavelengths that are distinct from the donor and acceptor, depending on the conformational change between the active and inactive states of the studied protein [4, 5] (**Fig 1A**). Thus, multiple-channel fluorescence images from the donor and the acceptor of the biosensor provide information about the activity of the studied protein.

FRET efficiency can be determined from the lifetime of the donor fluorophore, which requires special instruments in addition to wide-field or confocal fluorescence microscopes. For simple assessment of the FRET efficiency, fluorescence intensity-based FRET measures have been extensively proposed. The majority of the existing measures are based on three fluorescence images, *i*.*e*., fluorescence from the donor at the donor excitation, fluorescence from the acceptor at the donor excitation and fluorescence from the acceptor at the acceptor excitation [6–18]. However, in order to minimize photobleaching and specimen damage, as well as due to the simplicity of the imaging, ratiometric measures has been widely used based on two fluorescence images at the donor excitation; this measure just uses the ratio of the fluorescence intensity of the acceptor to that of the donor [19–21]. This ratiometric measure has advantages such as sensitivity to small changes in molecular activity and insensitivity to variations in the expression level of the FRET biosensor. However, the mechanism by which the ratiometric measure reflects actual biochemical processes has not been well discussed.

From the viewpoint of biochemical processes, the changing rate of the fraction of downstream active molecules is driven by the fraction of upstream active molecules, according to the law of mass action. Thus, the fraction of active molecules must be extracted from the FRET imaging. However, the ratiometric measure depends non-linearly on the fraction of active protein. Therefore, a new measure that reflects the fraction of active protein in a linear fashion is highly desired.

There are also other problems inherent to the current FRET imaging system. The first one is bleed-through signals, which occur when the fluorescence spectra of the donor and the accepter are overlapped (**Fig 1B**) [22], resulting in the obtained images not being true images of the donor and accepter fluorescence but mixed images of the two signals. The second one is cross-excitation, which occurs when excitation light intended for the accepter can also excite the donor and induce fluorescence [23], suggesting that the donor’s fluorescence is a mixture of the fluorescence from FRET and that from cross-excitation. The ratiometric measure based on such signal mixing was considered a reason for the low signal-to-noise (S/N) ratio in FRET imaging. To overcome these defects, a new measure that is unaffected by signal mixing is required.

In this study, to quantitatively obtain molecular activity via two channel fluorescence images in FRET imaging, we proposed a new measure that shows a linear relationship with the fraction of the active protein by faithfully modeling the observation process of two fluorescence channels. As an extension of this measure, we developed another new measure that provides more highly contrasting images than the ratiometric measure.

## Results

### Model of biosensor kinetics and fluorescence observation

We supposed that the biosensor stays in either an active or an inactive state (**Fig 1A**). The donor fluorescence (*F*_*d*_) and the acceptor fluorescence (*F*_*a*_) at the donor excitation are observed as weighted linear summations of the inactive and active states (*S*_*inact*_ and *S*_*act*_):

$$F_{d}=c_{inact,d}S_{inact}+c_{act,\;d}S_{act},$$ (1)

$$F_{a}=c_{inact,a}S_{inact}+c_{act,a}S_{act},$$ (2)

where *c*_*j*,*k*_ indicates the efficacy of *S*_*j*_ to *F*_*k*_ (*j* ∈ {*inact*,*act*} and *k* ∈ {*d*,*a*}) and the amount of the expressed biosensor, *S*_*tot*_, is represented by *S*_*tot*_ = *S*_*inact*_+*S*_*act*_. Note that *c*_*act*,*d*_ and *c*_*inact*,*a*_ represent the effects of bleed-through and cross-excitation. The traditional ratiometric measure, *m*_*RAT*_, is then simply defined as

$$m_{RAT}=F_{a}/F_{d}.$$ (3)

### Phase space of *F*_*i*_ and *F*_*a*_

**Fig 2:**
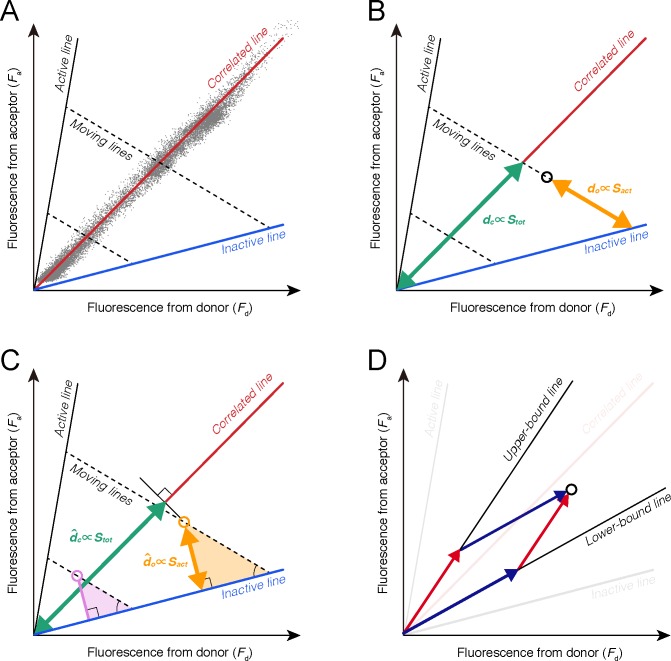
Phase space between the fluorescence from the donor and acceptor. **(A)** The inactive line (blue line) indicates states in which all biosensors are in the inactive state (*S*_*act*_ = 0). The correlated line (red line) indicates states in which average rates of back and forth reactions are balanced as *k*_*f*_(*t*)*S*_*act*_ = *k*_*b*_(*t*)*S*_*inact*_. The moving lines (black dotted arrows) indicate trajectories with varying *S*_*act*_. The gray scatter dots represent the typically observed relationship between *F*_*i*_ and *F*_*a*_ in the FRET imaging. **(B)** The lengths of the orange and purple arrows, *d*_*o*_ and *d*_*c*_, represent measures proportional to *S*_*act*_ and *S*_*tot*_. **(C)** The orange and pink right triangles have geometric similarity. The length of the perpendicular line, ${\hat{d}}_{o}$, is proportional to *d*_*o*_ and *S*_*act*_. The length of the purple arrows, ${\hat{d}}_{c}$, can be approximated when the state (*F*_*i*_, *F*_*a*_) is closely located along the correlated line. The LP measure is calculated by ${\hat{d}}_{o}/{\hat{d}}_{c}$. **(D)** Black lines were determined by the lower and upper ratiometric activity bounds. The states can be decomposed using two basis vectors along these black lines (dark blue and red arrows). The HC measure is calculated by a ratio between these factors.

As a preparation for the design of the new measures, we considered four unique lines in the phase space of *F*_*d*_ and *F*_*a*_. First, we defined the ‘*inactive line*’, along which all biosensor molecules remain in the inactive state with varying *S*_*tot*_, as *F*_*a*_ = *w*_*o*_*F*_*d*_ (blue line in **Fig 2A**). The ‘*active line*’, along which all biosensor molecules remain in the active state, can also be defined (black solid line in **Fig 2A**), although this line was not used in the design of the new measures. Next, we defined the ‘*moving line*’, along which an arbitrary state (*f*_*d*_, *f*_*a*_) moves in the phase space with a fixed *S*_*tot*_, as *F*_*a*_−*f*_*a*_ *= w*_*mov*_(*F*_*d*_−*f*_*d*_) (dashed lines in **Fig 2A**). The details of these linear functions with their parameter definitions are described in the **Materials and Methods** section. Finally, we defined the ‘*correlated line*’, along which *F*_*i*_ and *F*_*a*_ are distributed with positive correlation as linear function, *F*_*a*_ = *w*_*c*_*F*_*d*_ (red line and scatter plot in **Fig 1A**).

The slope of the inactive line, *w*_*o*_, can be approximately determined by imaging cells expressing only donor molecules with two channel wavelengths, because bleed-through from the acceptor emission (yellow line in **Fig 1B**) to the donor channel (cyan shaded region in **Fig 1B**) can be negligible in most circumstances, *i*.*e*., *c*_*act*,*d*_ = 0. The slope of the correlated line, *w*_*c*_, can be easily and practically determined by applying linear regression to the noisy observations.

### Linearly Proportional (LP) measure

The new quantity that we aim to measure is the fraction of active molecules. This value can be computed using the ratio of two independent variables *S*_*act*_ and *S*_*tot*_, which are the amount of active biosensor and the total amount of biosensor, respectively. In the phase space, we derived that the length of the moving line through an observed state (*F*_*d*_, *F*_*a*_) connecting the intersection with the inactive line is proportional to *S*_*act*_ (orange segment in **Fig 2B**; see Eq 11). In addition, we derived that the length of the intersection between an arbitrary zero-intercept line, e.g., the correlated line and the moving line through an observed state (*F*_*d*_, *F*_*a*_), *d*_*c*_, is proportional to *S*_*tot*_ (green segment in **Fig 2B**; see Eq 13) (see Materials and Methods). Consequently, *d*_*o*_/*d*_*c*_ can be a new measure that is linearly proportional to the fraction of active molecules and is unaffected by signal mixing due to bleed-through and cross-excitation.

Calculation of *d*_*o*_ and *d*_*c*_ requires the slope of the moving line, *w*_*mov*_, described by (*c*_*act*,*a*_−*c*_*inact*,*a*_)/(*c*_*act*,*d*_−*c*_*inact*,*d*_) (see Materials and Methods). However, it is technically difficult to independently quantify these four parameters (*c*_*inact*,*d*_, *c*_*inact*,*a*_, *c*_*act*,*d*_ and *c*_*act*,*a*_). We overcame the difficulty by focusing on alternative line segments, whose lengths are proportional to *d*_*o*_ and *d*_*c*_.

To compute *d*_*o*_, we set two arbitrary observation states and considered a right triangle consisting of the inactive line, the moving line going through each observation state, and an orthogonal line through the arbitrary state to the inactive line (orange and purple triangles in **Fig 2C**). Because of the geometric similarity between these triangles, the length of the orthogonal line segment, ${\hat{d}}_{o}$, is proportional to *d*_*o*_ (orange line segment in **Fig 2C**). Next, to compute *d*_*c*_, we focused on the property that *F*_*d*_ and *F*_*a*_ are distributed only in the vicinity of the correlated line (**Fig 2A**). Based on this property, *d*_*c*_ can be approximated by the length of an orthogonal projection of the observation state to the correlated line, ${\hat{d}}_{c}$ (green line segment in **Fig 2C**). Thus, the quantity almost proportional to *S*_*tot*_ can be measured by ${\hat{d}}_{c}$. In this way, a measure that is proportional to ${\hat{d}}_{o}/{\hat{d}}_{c}$ was computed by

$$m_{LP}(F_{d},F_{a})=\frac{F_{a}-w_{o}F_{d}}{F_{d}+w_{c}F_{a}}$$ (4)

(see Materials and Methods). This measure was called the ‘Linearly Proportional (LP) measure’.

We further examined how the LP measure is affected by the inevitable noise in the fluorescence emission and found that the LP measure is more robust than the ratiometric measure in most cases, namely, $w_{o}<w_{c}^{3}/(2w_{c}^{2}+1)$ (see Materials and Methods).

### Highly contrasting (HC) measure

The ratiometric measure can be rewritten as a non-linear function of the LP measure, *i*.*e*., the fraction of active molecules, as

$$m_{RAT}\propto \frac{m_{LP}+w_{1}}{w_{2}-m_{LP}},$$ (5)

where *w*_*1*_ = *w*_*o*_ and *w*_*2*_ = 1/*w*_*c*_. This relationship motivated us to propose another measure that maximizes the relative sensitivity to change in the LP measure. We then optimized *w*_*1*_ and *w*_*2*_, with the result that ${\hat{w}}_{1}=-\min(m_{LP})$ and ${\hat{w}}_{2}=\max(m_{LP})$. By substitution of Eq 4 into the optimized measures, we proposed the following new measure as

$$m_{HC}(F_{d},F_{a})=\frac{F_{a}-w_{l}F_{d}}{w_{u}F_{d}-F_{a}},$$ (6)

where *w*_*l*_ and *w*_*u*_ represent the maximum and minimum values of *m*_*RAT*_, respectively. This measure was called the ‘highly contrasting (HC) measure’ (the detailed derivation can be seen in the Materials and Methods). The HC measure corresponds to the ratiometric measure after decomposition with basis vectors, which represent the upper and lower bounds of the molecular activity (**Fig 2D**) (see Materials and Methods). To avoid divergence at *F*_*a*_/*F*_*d*_ = *w*_*u*_ in practice, the HC measure is modified to

$$m_{HC}(F_{d},F_{a})=\frac{F_{a}-w_{l}F_{d}}{(w_{u}+\alpha )F_{d}-F_{a}},$$ (7)

where *α* represents a small positive constant.

### Applications to artificial data and FRET imaging data

**Fig 3:**
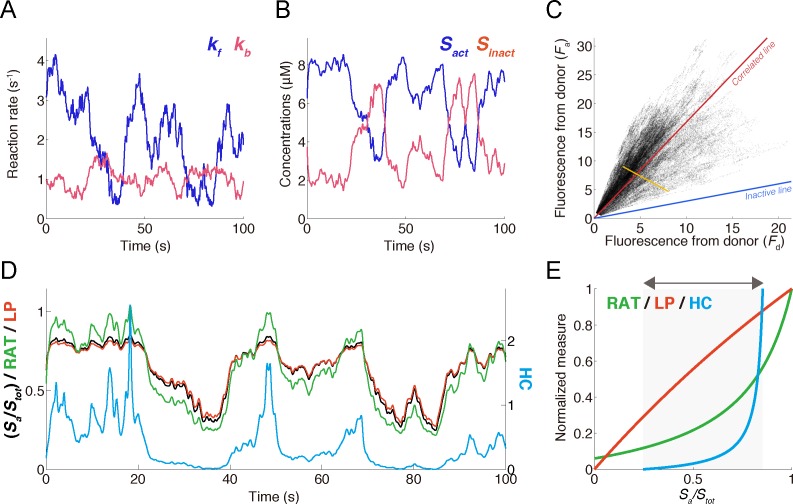
Application of the measures to artificially generated data. **(A)** In a simulation, the reaction rates of the biosensor, *k*_*f*_(*t*) and *k*_*b*_(*t*), fluctuated in time. These data were generated by an OU process.**(B)** Simulation result of *S*_*act*_ and *S*_*inact*_. **(C)** *S*_*act*_ and *S*_*inact*_ in (A) were converted to *F*_*a*_ and *F*_*i*_ in an observation process through two fluorescence channels with the bleed-through (Eqs 1 and 2) and indicated by the yellow line. The gray scatter dots indicate the distribution of *F*_*a*_ and *F*_*i*_, obtained by iterating the simulations and varying total amount of biosensor, *S*_*tot*_. The blue and red lines indicate the inactive line and the correlated line. **(D)** The black markings indicate the grand truth of *S*_*act*_/*S*_*tot*_, whereas the green, red and blue lines indicate the ratiometric, LP and HC measures, respectively. The amplitudes of these measures are normalized to minimize square errors from *S*_*act*_/*S*_*tot*_. **(E)** The dependencies of the ratiometric, LP and HC measures on *S*_*act*_/*S*_*tot*_. The HC measure responded within a dynamic range of *S*_*act*_/*S*_*tot*_ in (D), which is indicated by the black arrow and shaded region. Each measure was normalized so that the maximum value is 1.

We examined the characteristics of the LP and HC measures by using an artificially generated dataset. We simulated *S*_*inact*_ and *S*_*act*_ using the first-order reaction of the biosensor, in which forward and backward reaction rates were regulated by the time-dependent upstream signals (**Fig 3A and 3B**) (see Materials and Methods). *F*_*d*_ and *F*_*a*_ were calculated by mixing *S*_*inact*_ and *S*_*act*_ and taking the bleed-through effect into account (Eqs 1 and 2) (**Fig 3C**). The correlated line was calculated, without information about *k*_*f*_(*t*) or *k*_*b*_(*t*), from a joint distribution of *F*_*d*_ and *F*_*a*_, which was generated by independent simulations with a variety of *S*_*tot*_ values.

We calculated the LP, HC and ratiometric measures using the artificial *F*_*d*_ and *F*_*a*_ data. As expected, the LP measure followed *S*_*act*_/*S*_*tot*_ (red line in **Fig 3D**) well, whereas the ratiometric measure showed substantial deviation from *S*_*act*_/*S*_*tot*_ (green line in **Fig 3D**). On the other hand, the HC measure exhibited substantial underestimation but still showed high contrast for the temporal behaviors of *S*_*act*_/*S*_*tot*_ (blue line in **Fig 3D**). These characteristics are due to the linear and non-linear dependencies of the LP and HC measures on *S*_*act*_/*S*_*tot*_ (**Fig 3E**). Thus, the LP measure quantitatively provided the fraction of active molecules, while the HC measure qualitatively contrasted the temporal changes in *S*_*act*_/*S*_*tot*_. In addition, this simulation validated our approximation of the correlated line for the LP measure.

**Fig 4:**
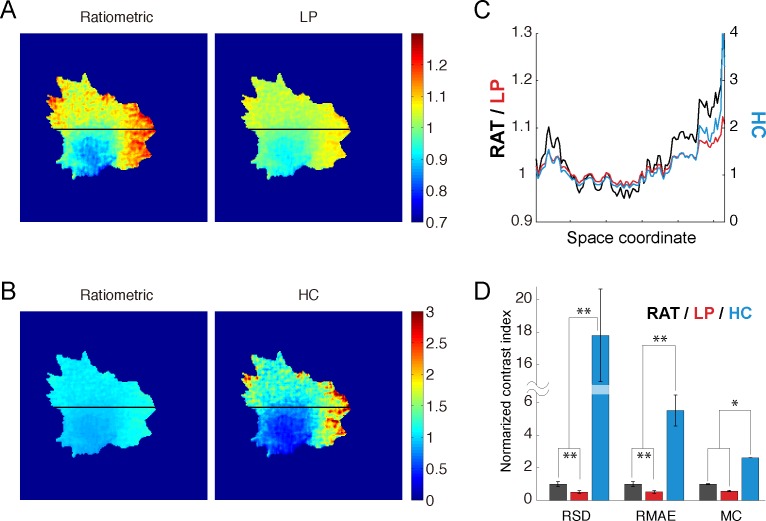
Experimental data of FRET imaging. **(A, B)** Snapshot images of the migrating cell expressing the biosensor Raichu–Cdc42. Images of the ratiometric measures are presented with those of the LP (A) and HC (B) measures in heat maps with the same range for comparison. **(C)** Intracellular distributions of the ratiometric, LP and HC measures along the black lines in A and B. **(D)** Image contrast was quantitatively evaluated using relative standard deviation (RSD), relative mean absolute error (RMAE), and Michelson contrast (MC). The error bars indicate the standard error. Significant differences are indicated (**p<0.01, *p<0.03; Mann-Whitney U test). These contrast measures at individual samples (cells) were provided in S1 Table.

We finally applied the LP and HC measures to FRET imaging. The activity of Cdc42 was monitored using FRET time-lapse imaging in spontaneously migrating human HT-1080 cells. For the LP measure, *w*_*o*_ was determined beforehand (see **S1 Fig**). **Fig 4A and 4B** present images of Cdc42 activity obtained using the ratiometric, LP and HC measures (**S1 Movie**). The images from the LP and HC measures were smoother and more highly contrasted than those from the ratiometric measure (**Fig 4A–4C**); these visual inspections were quantitatively evaluated using several indices: the relative standard deviation (RSD), Michelson contrast and relative mean absolute error (RMAE) (**Fig 4D**) (see **Materials and Methods**).

## Discussion

We proposed two new measures for FRET imaging, the LP and HC measures, which can be utilized for different purposes. The former is for quantifying active molecule fractions, and the latter is for obtaining maximal contrast images of molecular activity. These measures have good properties and preserve the basic characteristics of the ratiometric measure; the LP and HC measures are insensitive to the expression level of the biosensor and, thus, tolerant to its variability and also depend on the ratio of *F*_*a*_ to *F*_*d*_. We discuss the fundamental differences from the ratiometric measure as below.

### Properties of the LP measure

Although the LP measure was derived based on the observation process of two fluorescence channels, it can be applicable to any single molecular FRET biosensor measurement without knowledge of the bleed-through of the observation process. In addition, the LP measure has several favorable characteristics. First, the LP measure is linearly proportional to the fraction of active molecules, which is in contrast to the non-linear dependency of the ratiometric measure (**Fig 3E**). Thus, the LP measure can be used to estimate the concentration of active molecules if the biosensors are uniformly distributed within specific subcellular regions, such as the cytosol, nucleus or membrane, and imaged using a scanning microscopy technique, such as confocal microscopy or two-photon excitation microscopy [24, 25]. Second, the LP measure naturally has an unmixing character even from mixed images with bleed-through signals. Third, the LP measure is more robust against inevitable noise in fluorescence than the ratiometric measure.

### Properties of the HC measure

The HC measure showed vividly contrasted images of the molecular activity (**Fig 4B**) because it was designed by optimizing the relative sensitivity to changes in the fraction of active molecule. Although the HC measure does not necessarily reflect the physical or chemical meanings like the ratiometric measure, but unlike the LP measure, the HC measure could be a more useful tool for the visualization of high-contrast images than the ratiometric measure. In addition, the HC measure geometrically represents the decomposition of a state vector into two basis vectors in the phase space. These vectors represent the upper and lower bounds of the ratiometric activity (**Fig 2D**). The HC measure corresponds to the ratio between the factors with respect to the two basis vectors (see Materials and Methods).

### Computational approaches with the LP measure

Several computational studies have performed simulations of intracellular signaling to reproduce molecular activity time series for comparison with FRET imaging [26–30]. Because the mathematical models in these studies usually describe changes in the concentrations of signaling molecules, the LP measure should be useful for comparisons of simulations and experiments. In the computational data analyses of FRET imaging, dynamical relationships between molecular activity and certain biological outputs, e.g., membrane protrusion in our example (**Fig 4**), have been examined via cross-correlation analysis [27, 31, 32], and intracellular signal transfer from the molecular activity to the biological output has been identified as the response function [33, 34]. In these analyses, the LP measure could be favorable for avoiding signals that are biasedly amplified by the ratiometric measure (**Fig 3D**).

## Materials and Methods

### Inactive, moving and correlated lines

The inactive line, along which all biosensors remain in the inactive state, is derived from Eqs 1 and 2 when *S*_*inact*_ = *S*_*tot*_ and *S*_*act*_ = 0 by

$$F_{a}=w_{o}F_{d},$$ (8)

where *w*_*o*_ = *c*_inact,a_/*c*_inact,d_.

The moving line represents the trajectory in phase space from an arbitrary state (*f*_*d*_, *f*_*a*_) with varying *S*_*act*_ that is subject to constant *S*_*tot*_, and thus, its function is expressed by

$$F_{a}-f_{a}=w_{mov}(F_{d}-f_{d}),$$ (9)

where *w*_*mov*_ = *dF*_*a*_/*dF*_*d*_ = (*dF*_*a*_/*dS*_*act*_)/(*dF*_*d*_/*dS*_*act*_) *=* (*c*_*act*,*a*_−*c*_*inact*,*a*_)/(*c*_*act*,*d*_−*c*_*inact*,*d*_), which is negative constant.

### Line segments that represent *S*_*act*_ and *S*_*tot*_

In the phase space (**Fig 2B**), the line segment from an observed state (*F*_*d*_, *F*_*a*_) to the intersection between the inactive line and the moving line through (*F*_*d*_, *F*_*a*_). Its length is calculated from Eqs 8 and 9 as

$$\begin{array}{l} d_{o}=\sqrt{{\left(F_{d}-\frac{F_{a}-w_{mov}f_{d}}{w_{o}-w_{mov}}\right)}^{2}+{\left(F_{a}-w_{o}\frac{F_{a}-w_{mov}F_{d}}{w_{o}-w_{mov}}\right)}^{2}}\\ \;=\frac{\sqrt{1+w_{mov}^{2}}}{w_{o}-w_{mov}}\left(w_{o}F_{d}-F_{a}\right) \end{array}.$$ (10)

Then, substitutions of *w*_*mov*_, *w*_*o*_, *F*_*d*_ and *F*_*a*_ (Eqs 1 and 2) lead to

$$d_{o}=\sqrt{{\left(c_{inact,d}-c_{act,d}\right)}^{2}+{\left(c_{act,a}-c_{inact,a}\right)}^{2}}S_{a}.$$ (11)

The length of the line segment from (0, 0) to the intersection between an arbitrary zero-intercept line, i.e., *F*_*a*_ = *w*_*any*_*F*_*d*_ (*w*_*any*_ is arbitrary) and the moving line through the observed state (*F*_*d*_, *F*_*a*_) is calculated from Eq 9 as

$$\begin{array}{l} d_{c}=\sqrt{{\left(\frac{F_{a}-w_{mov}F_{d}}{w_{any}-w_{mov}}\right)}^{2}+{\left(w_{any}\frac{F_{a}-w_{mov}F_{d}}{w_{any}-w_{mov}}\right)}^{2}}\\ \;=\frac{\sqrt{1+w_{any}^{2}}}{w_{any}-w_{mov}}\left(F_{a}-w_{mov}F_{d}\right) \end{array}.$$ (12)

Then, substitutions of *F*_*d*_ and *F*_*a*_ (Eqs 1 and 2) lead to

$$d_{c}=\frac{\sqrt{1+w_{any}^{2}}}{w_{any}-w_{mov}}\left(c_{inact,a}-w_{mov}c_{inact,d}\right)S_{tot}.$$ (13)

Note that because *w*_*any*_ is arbitrary, *w*_*any*_ can be replaced by *w*_*c*_.

### Derivation of the LP measure

Due to the geometric similarity between the two right triangles shown in **Fig 2C**, *d*_*o*_ is proportional to the length of the perpendicular line segment from the observed state (*F*_*d*_, *F*_*a*_) to the inactive line, ${\hat{d}}_{o}$ (orange line in **Fig 2C**), which is calculated as

$${\hat{d}}_{o}=\left(F_{a}-w_{o}F_{d}\right)/\sqrt{1+w_{o}^{2}}.$$ (14)

Because joint distribution of *F*_*d*_ and *F*_*a*_ was restricted in the vicinity of the correlated line (**Fig 2A**), we approximated *d*_*c*_ using the Euclidean norm of the orthogonally projected point from the observed state (*F*_*d*_, *F*_*a*_) to the correlated line, ${\hat{d}}_{c}$ (green line in **Fig 2C**), which is calculated as

$${\hat{d}}_{c}=\left(F_{d}+w_{c}F_{a}\right)/\sqrt{1+w_{c}^{2}}.$$ (15)

Therefore, the LP measure can be computed by

$$m_{LP}≃\frac{{\hat{d}}_{o}}{{\hat{d}}_{c}}\propto \frac{F_{a}-w_{o}F_{d}}{F_{d}+w_{c}F_{a}}.$$ (16)

### Reliability of the LP measure

We examined the robustness of the LP measure against fluorescence noise, where photons are randomly emitted from the fluorophore via a Poisson process according to physical law [35]. The observation process was described by

$$F_{d}={\bar{F}}_{d}+\varepsilon_{d},$$ (17)

$$F_{a}={\bar{F}}_{a}+\varepsilon_{a},$$ (18)

where ${\bar{F}}_{d}$ and ${\bar{F}}_{a}$ indicate averages of *F*_*d*_ and *F*_*a*_, respectively, and *ε*_*d*_ and *ε*_*a*_ indicates deviations from their averages. These deviations obey a normal distribution with 0 mean; the variances of *ε*_*d*_ and *ε*_*a*_ are ${\bar{F}}_{d}$ and ${\bar{F}}_{a}$, respectively. This is due to the nature of Poisson processes; the variance of the number of emitted photons equals its average if the average is enough large. In this setting, we approximately calculated the LP measure as

$$m_{LP}≃\frac{{\bar{F}}_{a}-w_{o}{\bar{F}}_{d}}{{\bar{F}}_{d}+w_{c}{\bar{F}}_{a}}+\frac{1+w_{c}w_{o}}{{\left({\bar{F}}_{d}+w_{c}{\bar{F}}_{a}\right)}^{2}}\left({\bar{F}}_{d}\varepsilon_{a}-{\bar{F}}_{a}\varepsilon_{d}\right)$$ (19)

by the first-order Taylor expansion around ${\bar{F}}_{d}$ and ${\bar{F}}_{a}$, where the first term represents the average of *m*_*LP*_, ${\bar{m}}_{LP}$. The standard deviation of *m*_*LP*_ is calculated as

$$SD(m_{LP})=\frac{1+w_{c}w_{o}}{{\left({\bar{F}}_{d}+w_{c}{\bar{F}}_{a}\right)}^{2}}\sqrt{{\bar{F}}_{d}^{2}{\bar{F}}_{a}+{\bar{F}}_{a}^{2}{\bar{F}}_{d}}.$$ (20)

Here, we defined the reliability of the LP measure by $SD(m_{LP})/{\bar{m}}_{LP}$ at the correlated line along which most of states (*F*_*d*_, *F*_*a*_) are restricted, ${\bar{F}}_{a}=w_{c}{\bar{F}}_{d}$ (**Fig 2A**). Note that the lower this value is, the more reliable the LP measure becomes. The reliability of the ratiometric measure, $SD(m_{RAT})/{\bar{m}}_{RAT}$, was also calculated in the same way. We then evaluated the reliability of the LP measure compared with the ratiometric measure by

$$\frac{SD(m_{LP})}{{\bar{m}}_{LP}}/\frac{SD(m_{RAT})}{{\bar{m}}_{RAT}}=w_{c}\frac{1+w_{c}w_{o}}{\left(1+w_{c}^{2})(w_{c}-w_{o}\right)}.$$ (21)

Therefore, the LP measure has better reliability than the ratiometric measure, if

$$w_{o}<\frac{w_{c}^{3}}{2w_{c}^{2}+1}.$$ (22)

Most FRET imaging satisfies this inequality because *w*_*c*_ is usually much larger than *w*_*o*_.

### Derivation of the HC measure

We consider a general form of the measure as

$$m_{OP}(m_{LP}|w_{1},w_{2})=\frac{m_{LP}+w_{1}}{w_{2}-m_{LP}},$$ (23)

where *w*_*1*_ and *w*_*2*_ indicate constant parameters. For the best visual appearance, we defined the goodness of the measure by the relative sensitivity to change integrated over its dynamics range:

$$U(w_{1},w_{2})=\int_{\min(m_{LP})}^{\max(m_{LP})}\frac{d}{dm_{LP}}m_{OP}(m_{LP}|w_{1},w_{2})dm_{LP},$$ (24)

where max(*m*_*LP*_) and min(*m*_*LP*_) describe the maximum and minimum values, respectively, of the LP measure in the time-lapse FRET images. The greater the goodness, the better the generated image. Then, we optimized the parameters, *w*_*1*_ and *w*_*2*_, to maximize *U* as

$$\left({\hat{w}}_{1},{\hat{w}}_{2}\right)=\underset{w_{1},w_{2}}{\arg\;\max}\;U(w_{1},w_{2}),$$ (25)

Through this optimization, we obtained ${\hat{w}}_{1}=-\min(m_{LP})$ and ${\hat{w}}_{2}=\max(m_{LP})$. Thus, we proposed the HC measure as

$$m_{HC}(m_{LP})=\frac{m_{LP}-\min(m_{LP})}{\max(m_{LP})-m_{LP}}.$$ (26)

By substitution of Eq 4 to Eq 26, we found the fluorescence-based HC measure as

$$m_{HC}(F_{d},F_{a})=\frac{F_{a}-w_{l}F_{d}}{w_{u}F_{d}-F_{a}},$$ (27)

where *w*_*l*_ and *w*_*u*_ represents min(*m*_*RAT*_) and max(*m*_*RAT*_), respectively.

### Relationship of the HC measure to vector decomposition

In the phase space, we consider vector decomposition of an arbitrary state **f** = (*F*_*d*_, *F*_*a*_)^T^ into two basic unit vectors, **e**_*u*_ = (1,*w*_*u*_)^T^/(1+*w*_*u*_^2^)^1/2^ and **e**_*l*_ = (1,*w*_*l*_)^T^/(1+*w*_*l*_^2^)^1/2^, as

f=Eq, (28)

where **E** = (**e**_*l*_, **e**_*u*_) and **q** = (*q*_*l*_, *q*_*u*_)^T^ (**Fig 2D**). *q*_*l*_ and *q*_*u*_ can be obtained by multiplying **E**^-1^. Then, *q*_*l*_ and *q*_*u*_ are found to be proportional to *w*_*u*_*F*_*d*_−*F*_*a*_ and *F*_*a*_−*w*_*l*_*F*_*d*_, which are identical to the denominator and the numerator of the HC measure, respectively. Thus, the HC measure substantively represents the ratiometry in the linear span of **e**_*u*_ and **e**_*l*_.

### Simulation of artificial data

We generated the artificial data by simulating the first-order reaction of the biosensor as

$${\text{S}}_{inact}\overset{k_{f}(t)}{\underset{k_{b}(t)}{⇄}}{\text{S}}_{act},$$ (29)

where S_inact_ and S_act_ indicate the inactive and active states, respectively; *k*_*f*_ (*t*) and *k*_*b*_(*t*) indicate the rates of the forward and backward reactions, respectively, which are regulated by time-dependent upstream signals. This dynamics was described by the ordinary differential equation:

$$\frac{dS_{a}}{dt}=k_{f}(t)(S_{tot}-S_{a})-k_{b}(t)S_{a},$$ (30)

where *k*_*f*_(*t*) and *k*_*b*_(*t*) follow a stochastic process called the Ornstein-Uhlenbeck process:

$$\frac{dk_{i}}{dt}=-\mu_{i}(k_{i}-ko_{i})+\beta_{i}\xi_{i},$$ (31)

where *μ*_*i*_, *ko*_*i*_ and *β*_*i*_ indicate positive constants and *i* ∈ {*f*,*b*}. Through Eqs 1 and 2, *F*_*d*_ and *F*_*a*_ are calculated as the observed fluorescences of the donor and the acceptor. The parameters used in **Fig 3**are the following: *S*_*tot*_ = 10; *μ*_*f*_ = 0.002; *ko*_*f*_ = 3; *β*_*f*_ = 0.4; *μ*_*b*_ = 0.004; *ko*_*f*_ = 1; *β*_*f*_ = 0.15; *c*_*i*,*i*_ = 1; *c*_*a*,*a*_ = 1; *c*_*i*,*a*_ = 0.3; *c*_*a*,*i*_ = 0.2.

### Indexes of the image contrast

In **Fig 4A and 4B**, we quantitatively evaluated the image contrast using the following indexes:

Relative standard deviation (RSD):

$$RSD=\frac{1}{\bar{I}(N-1)}\sum_{j}^{T}\sum_{i\in Q_{j}}{\left(I_{ij}-\bar{I}\right)}^{2},$$ (32)

where *T*, *I*_*ij*_, and *Q*_*j*_ indicate the number of frames, the intensity of the *i*th pixel in the *j*th frame, and the set of pixels within the intracellular region in the *j*th frame, respectively; $\bar{I}$ and *N* indicate the mean of the pixel intensities within the intracellular regions of all frames and the number of pixels within the intracellular regions of all frames, respectively:

$$\bar{I}=\frac{1}{N}\sum_{j}^{T}\sum_{i\in Q_{j}}I_{ij},$$ (33)

$$N=\sum_{j}^{T}\sum_{i\in Q_{j}}1.$$ (34)

Relative mean absolute error (RMAE):

$$RMAE=\frac{1}{\bar{I}N}\sum_{j}^{T}\sum_{i\in Q_{j}}\left|I_{i}-\bar{I}\right|.$$ (35)

Michelson contrast (MC) [36]:

$$MC=\frac{\max(I)-\min(I)}{\max(I)+\min(I)}.$$ (36)

where max(.) and min(.) are operations that return the maximum and minimum pixel intensities within the intracellular region.

### Cells, Plasmids, and Reagents

HT-1080 cells were purchased from the American Type Culture Collection or the Japan Cell Resource Bank and maintained in Dulbecco’s modified Eagle’s medium (DMEM) (Sigma-Aldrich, St. Louis, MO) supplemented with 10% fetal bovine serum (FBS). The cells were transfected with the plasmid encoding the FRET biosensors, Raichu-Cdc42/1054x [37] with Lipofectamine 2000 reagent according to the manufacturer’s protocol (Invitrogen, San Diego, CA). HeLa cells were purchased from the Human Science Research Resources Bank (Sennanshi, Japan) and maintained in DMEM (Sigma-Aldrich, St. Louis, MO) supplemented with 10% fetal bovine serum.

### Time-lapse FRET Imaging

FRET imaging of the Cdc42 activity in randomly migrating HT-1080 cells was performed as described previously [27, 38]. Briefly, HT-1080 cells expressing FRET biosensors were suspended using trypsin, plated on collagen-coated 35-mm glass-bottomed dishes, and cultured with Phenol Red-free DMEM/F12 (Invitrogen) containing 10% FBS for approximately 1 hour. Prior to imaging, the culture medium was overlaid with mineral oil to prevent evaporation. The cells were imaged with an inverted microscope (IX81; Olympus, Tokyo, Japan) equipped with an UPLANSAPO 60x oil-immersion objective lens (Olympus), a cooled charge-coupled device (CCD) camera (Cool SNAP-K4; Roper Scientific), a light-emitting diode (LED) illumination system (CoolLED precisExcite, Molecular Devices), and an IX2-ZDC laser-based autofocusing system (Olympus) with an MDXY30100T-Meta automatically programmable XY stage (Sigma Koki, Tokyo, Japan). The following filters were used for the dual-emission imaging studies: excitation filters, 435/20 for cyan fluorescent protein (CFP) and FRET (Olympus); dichroic mirrors, XF2034 (Omega); and emission filters, 480AF30 for CFP (Omega) and 535AF26 for FRET (Omega). The exposure time was 200–400 milliseconds for CFP and the FRET images when the binning was set to 4x4.

### Determination of w_o_ for the LP measure

For imaging, HeLa cells were seeded on a 35-mm glass-base dish (Asahi Techno Glass, Tokyo, Japan), and transfected pCAGGS-FLAG-AktPH-mCFP [39] using 293fectin (Invitrogen, San Diego, CA) according to the manufactures’ instructions. Two days after transfection, the medium was replaced with FluoroBrite (Invitrogen) supplemented with GlutamMax (Invitrogen) and 0.1% bovine serum albumin. Several hours after medium change, the cell were imaged under the same FRET imaging conditions (S1 **Fig**).

### Cell edge detection

The cellular edges were extracted from the CFP image. The CFP image was first deblurred via blind image deconvolution [40], in which the original image was estimated using a maximum likelihood method without explicit knowledge of the point spread function in the microscopy. The deblurred CFP image was then smoothed using a Savitzky-Golay filter, which smoothed each of the local image patches (5x5) with a second-order polynomial smoothing kernel. This preprocessing was beneficial for improving the accuracy of subsequent cellular edge detections. The smoothed CFP images were then binarized with a certain threshold, which was tuned so that intracellular and extracellular regions were clearly segregated. Each pixel in the CFP image was classified into either a high-intensity or a low-intensity pixel, which corresponded to the intra- and extracellular regions, respectively. The threshold value was set so that it was the same over the space and time-lapses for each cell. The small hole-like extracellular regions surrounded by intracellular regions were filled with the intracellular regions. Finally, the cellular edge was obtained as a closed loop of the intracellular region.

## Supporting Information

S1 Fig Determination of *w*_*o*_ for the LP measure. **(A)** HeLa cells expressing Akt-PH-mCFP were imaged using the channels for CFP fluorescence (475 nm) and YFP fluorescence (530 nm). Circles indicate regions of interest (ROIs) for quantification. **(B)** The relationship between the intensities of the CFP and the YFP channels within the ROIs shown in (A) was plotted. This slope approximately corresponds to *w*_*o*_ for the LP measure (*w*_*o*_ = 0.373).
(TIFF)

S1 Movie The ratiometric, LP and HC measures of time-lapse FRET imaging. Movies of the ratiometric, LP and HC measures in the migrating cell expressing the biosensor Raichu–Cdc42. The ratiometric measures (RAT) are presented with those of the LP and HC measures in heat maps with the same range for comparison.
(MOV)

S1 Table Individual data points in Fig 4D. Values of RSD, RMAE and MC at individual samples (cells) are presented.
(TIFF)
