# Supplementary figures and images for: Two New FRET Imaging Measures: Linearly Proportional to and Highly Contrasting the Fraction of Active Molecules
Source: PLoS One. 2016 Oct 25;11(10):e0164254. doi: 10.1371/journal.pone.0164254 (PMC5079603; doi:10.1371/journal.pone.0164254)

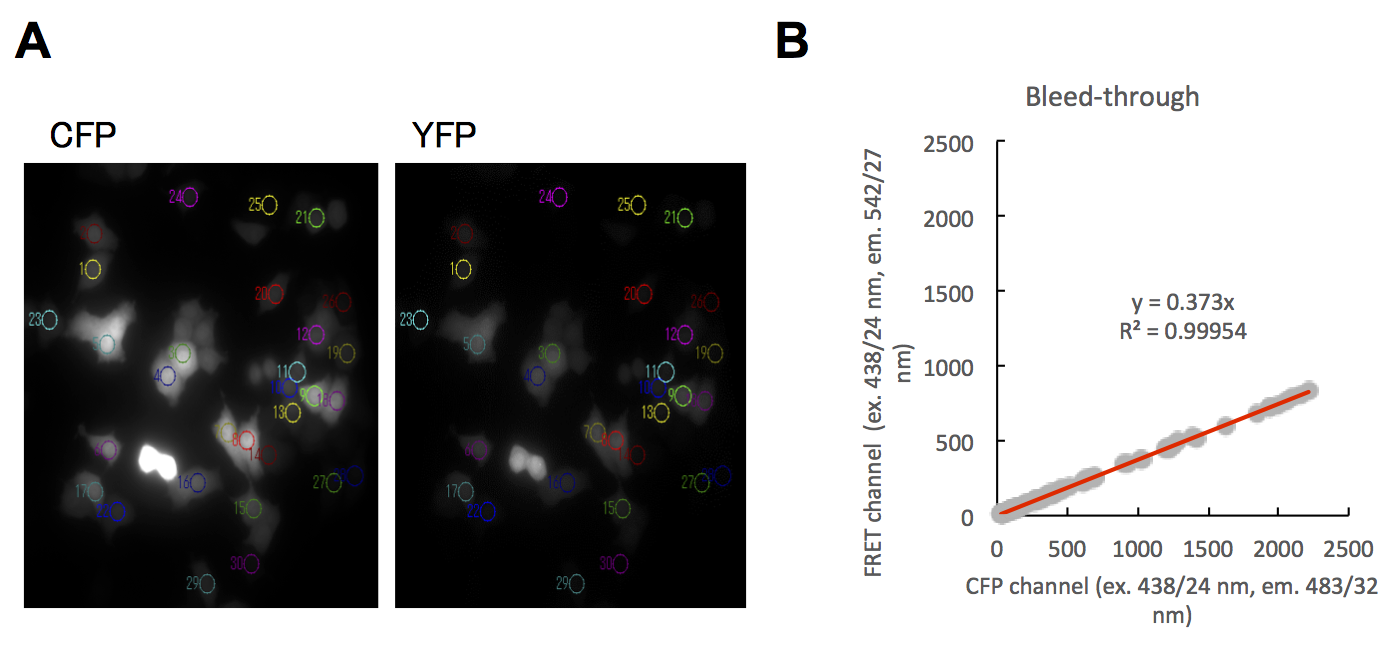

Supplement: S1 Fig — (A) HeLa cells expressing Akt-PH-mCFP were imaged using the channels for CFP fluorescence (475 nm) and YFP fluorescence (530 nm). Circles indicate regions of interest (ROIs) for quantification. (B) The relationship between the intensities of the CFP and the YFP channels within the ROIs shown in (A) was plotted. This slope approximately corresponds to wo for the LP measure (wo = 0.373). (TIFF) [file pone.0164254.s001.tiff]

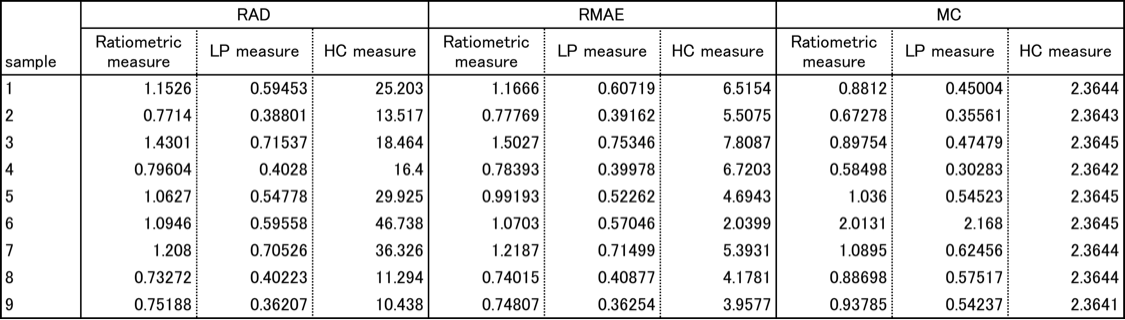

Supplement: S1 Table — Values of RSD, RMAE and MC at individual samples (cells) are presented. (TIFF) [file pone.0164254.s003.tiff]
